# Global trends and hotspots related to whiplash injury: A visualization study

**DOI:** 10.1097/MD.0000000000038777

**Published:** 2024-07-19

**Authors:** Yaqi He, Jinpeng Gao, Yang Liu, Jinghua Qian

**Affiliations:** a School of Sports Medicine and Rehabilitation, Beijing Sport University, Beijing, China.

**Keywords:** bibliometric analysis, global trends, visualization, whiplash injury

## Abstract

Whiplash injury, commonly occurring as a result of car accidents, represents a significant public health concern. However, to date, no comprehensive study has utilized bibliometric approaches to analyze all published research on whiplash injury. Therefore, our study aims to provide an overview of current trends and the global research landscape using bibliometrics and visualization software. We performed a bibliometric analysis of the data retrieved and extracted from the Web of Science Core Collection database in whiplash injury research up to December 31, 2022. Research articles were assessed for specific characteristics, such as year of publication, country/region, institution, author, journal, field of study, references, and keywords. We identified 1751 research articles in the analysis and observed a gradual growth in the number of publications and references. The United States (379 articles, 21.64%), Canada (309 articles, 17.65%), and Australia (280 articles, 16.00%) emerged as the top-contributing countries/regions. Among institutions, the University of Queensland (169 articles, 9.65%) and the University of Alberta (106 articles, 6.05%) demonstrated the highest productivity. “Whiplash,” “Neck Pain,” “Cervical Spine Disease,” and “Whiplash-associated Disorders” are high-frequency keywords. Furthermore, emerging areas of research interest included traumatic brain injury and mental health issues following whiplash injury. The number of papers and citations has increased significantly over the past 2 decades. Whiplash injury research is characteristically multidisciplinary in approach, involving the fields of rehabilitation, neuroscience, and spinal disciplines. By identifying current research trends, our study offers valuable insights to guide future research endeavors in this field.

## 1. Introduction

Whiplash injury refers to damage to the neck caused by the inertia force acting on the head during a motor vehicle collision, resulting in neck pain and injury to one or more segments of the cervical spine.^[1]^ Most individuals affected by whiplash injury commonly experience symptoms like neck pain and limited mobility. Additionally, some may develop chronic symptoms such as headaches, hearing loss, dizziness, tinnitus, memory loss, difficulty swallowing, temporomandibular joint pain, sensory dysfunction, and other persistent issues. These symptoms collectively constitute what is known as whiplash-associated disorders (WAD).^[2]^ Depending on the presence and severity of musculoskeletal and neurological symptoms, WAD can be classified into grades ranging from 0 to IV.^[3]^

Whiplash injury can result from various factors, and it does not always lead to pathological changes. However, when subjected to greater impact forces, it can cause immediate damage to structures such as intervertebral discs, ligaments, and paraspinal muscles. Additionally, brain injury is also a potential consequence following a whiplash injury.^[4]^

Integrated multimodal therapies consisting of pain neuroscience education, cognitively targeted exercise therapy, and stress management are now used in clinical practice to treat patients with WAD.^[5]^ Despite its negative reputation, WAD is generally considered a relatively benign injury, with most patients achieving full recovery.^[6]^ Studies indicate that 88% of patients achieve symptom resolution within 2 months, with 93% achieving complete recovery within 3 months. However, a small percentage of patients may continue to experience symptoms for up to 2 years, and in some cases, symptoms may persist for as long as 7 years.^[7,8]^ Whiplash injury is prevalent globally, with its incidence influenced by geographic distribution. In the United Kingdom, for instance, whiplash injury accounts for 76% of all insurance claims,^[9,10]^ and approximately $3.9 billion are spent annually in the United States on medical care.^[11]^ Understanding the current state of development is critical because of the enormous expenditure and high incidence of whiplash injury.

Bibliometrics is a quantitative approach used to describe and analyze the dynamics and progress within a specific field. This method enables the visualization of literature analysis results, aiding in data interpretation and providing easy access to a vast array of publications on a particular topic or area of study. It allows for the identification of influential publications, collaborations between countries, authors, institutions, and active journals.^[12]^ Currently, only one study has analyzed the heavily cited literature in the field of whiplash injury and its research characteristics.^[13]^ This study focused on the top 100 most cited documents, offering insights into the contributions of countries, organizations, and authors to the field. However, this analysis did not explore keywords and research trends related to whiplash injury. Moreover, the relatively small sample size used in this study may not fully capture the entirety of the field’s development.

To address this knowledge gap, our study utilized the Web of Science™ Core Collection (WoSCC). We conducted a comprehensive search of all literature on whiplash injury dating back to 1953 and performed descriptive statistics. The main objective of our study was to statistically identify publications and collaborations (involving countries, organizations, authors, and journals) in the field and to uncover research hotspots.

## 2. Methods

### 2.1. Search strategy

The WoSCC database is widely acknowledged as a premier data source and is considered the most suitable database for bibliometric research.^[14,15]^ To systematically retrieve relevant literature, a thorough search was conducted utilizing the Medical Subject Headings “Whiplash injury.” The final search strategy was as follows: TS = (whiplash injury) AND Document Type = (article) AND Language = (English), with the publication date set from January 1, 1953, to December 31, 2022.

### 2.2. Data collection

Data collection was conducted independently by 2 researchers (YH and JG), and any discrepancies were resolved through discussion with the senior researcher (JQ) for adjudication. The “All Record & Cited References” option was utilized to download data from the included articles in the WoSCC (Attachment 1, Supplemental Digital Content, http://www.w3.org/1999/xlink). This option provides access to 29 fields, and the data were exported in a custom “plain text document” format. Any missing data were manually supplemented from the original literature sources.

### 2.3. Statistical analysis

Co-occurrence (COOC, version 14.3) software was employed for bibliometric analysis and network visualization. To prepare the analyzed data, COOC was utilized to merge the downloaded txt files, eliminate duplicates, and convert the file format to xlsx (Attachment 2, Supplemental Digital Content, http://www.w3.org/1999/xlink). Synonyms for keywords were consolidated, and any missing information such as keywords or author affiliations was supplemented.^[16]^ Subsequently, VOSviewer (1.6.19), a software tailored for visualizing bibliometric networks, was utilized to examine collaborations among countries, institutions, and authors, as well as author co-citations and keyword co-occurrences.^[17]^ Moreover, CiteSpace (6.4.2 R4 64-bit Standard), a visual analytics program developed by Dr Chao-Mei Chen in Java, was utilized for co-citation analysis and mapping in knowledge domains.^[18]^ Specifically, CiteSpace was utilized for burstiness analysis of keywords.

## 3. Results

### 3.1. Study selection and data handling

**Figure 1.**
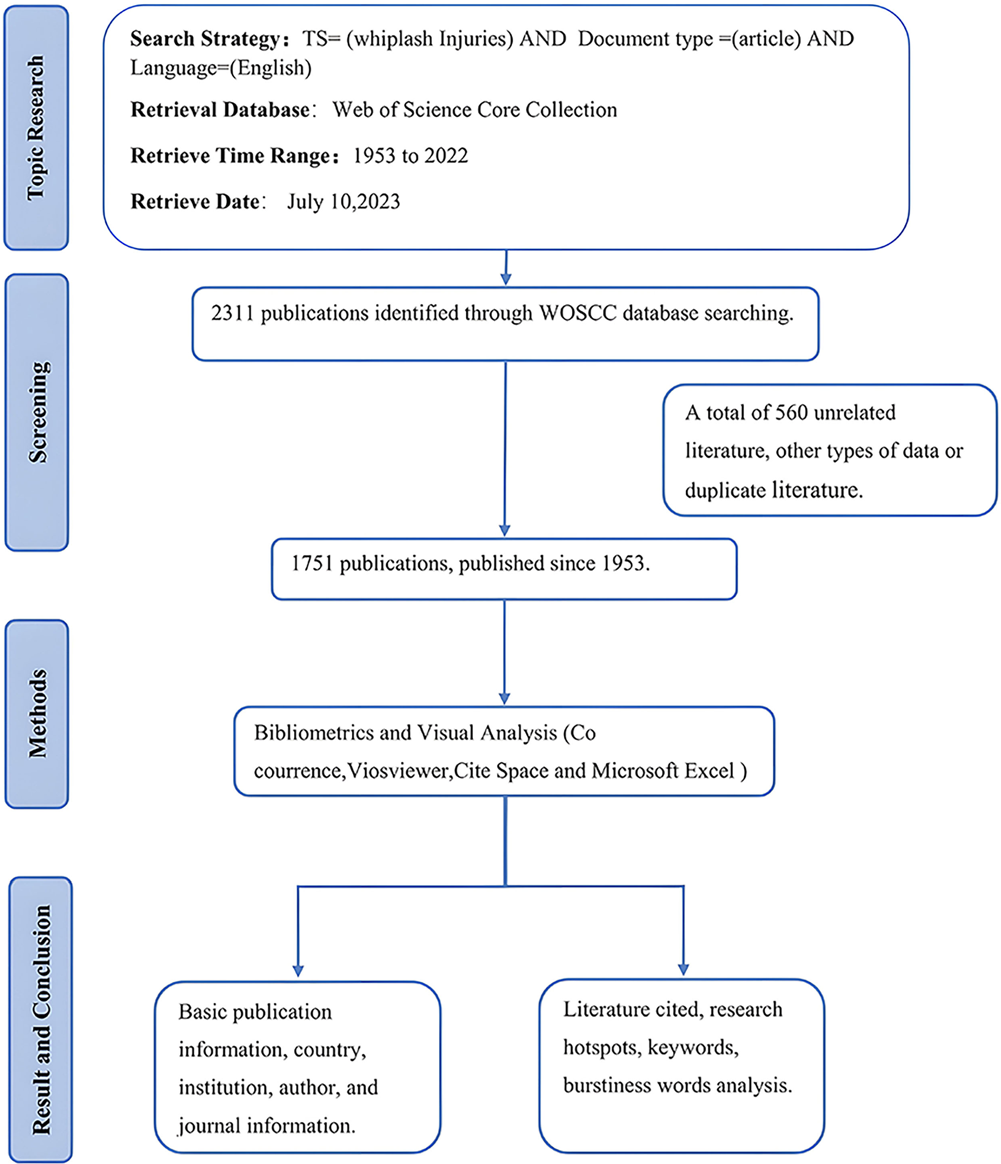
Flowchart.

Based on our search strategy, a total of 2311 articles were initially retrieved. Following independent screening by 2 researchers and subsequent discussions with the senior researcher, 1751 studies were retained (Fig. 1). A total of 395 fields for author keywords were missing, and after attempting to supplement them by copying plus keywords to the author keywords field, 66 missing fields remained. Therefore, these 66 articles were eliminated from the keyword analysis. A total of 3218 keywords were identified among 1685 studies after merging 956 synonyms and removing meaningless words by COOC. Additionally, we observed missing columns for 45 institutions and 42 countries/regions, which were completed based on the original documentation.

### 3.2. Global annual publications output analysis

**Figure 2.**
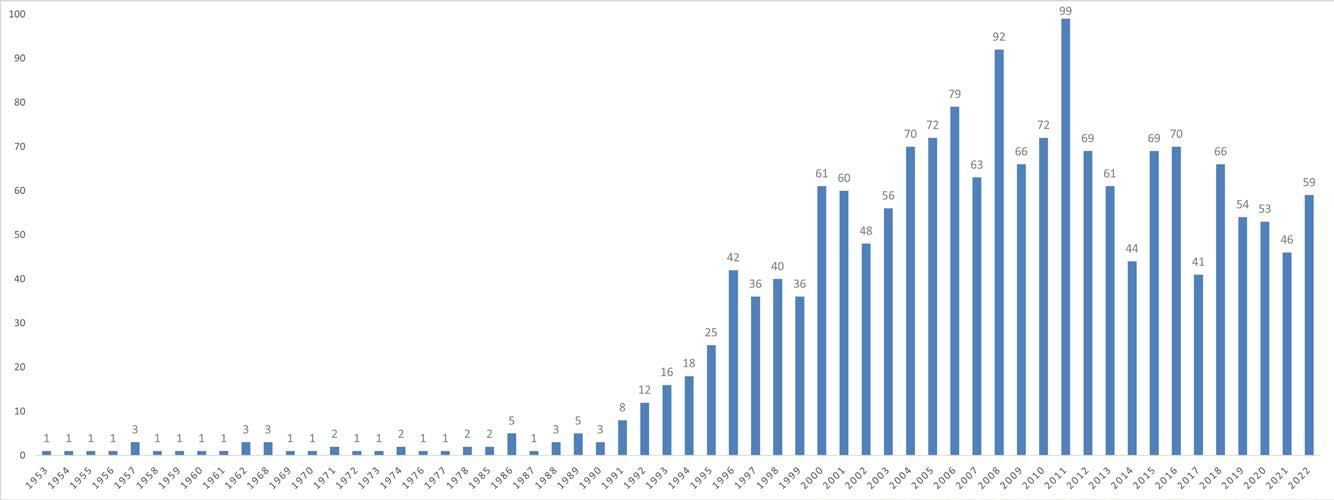
Number of publications in the field of whiplash injuries from 1991 to 2022.

The study encompassed a total of 1751 articles authored by 4269 authors affiliated with 1631 institutions spanning 57 countries. These articles were disseminated across 451 journals and collectively accrued 31,661 citations from 8662 journals. On average, each article received 18.08 citations. The trend in publication output regarding whiplash injury is illustrated in Figure 2. Before 1990, the annual publication rate remained modest, averaging 1.85 papers per year with minimal fluctuations. However, from 1991 to 2022, a significant surge in publications was observed, peaking at 99 papers in 2011. Throughout this period, an average of 51.84 papers were published annually. The global annual publication count surged from 3 papers in 1990 to 99 papers in 2011, marking a remarkable growth of 32 times.

### 3.3. Analysis of geographic distribution

**Figure 3.**
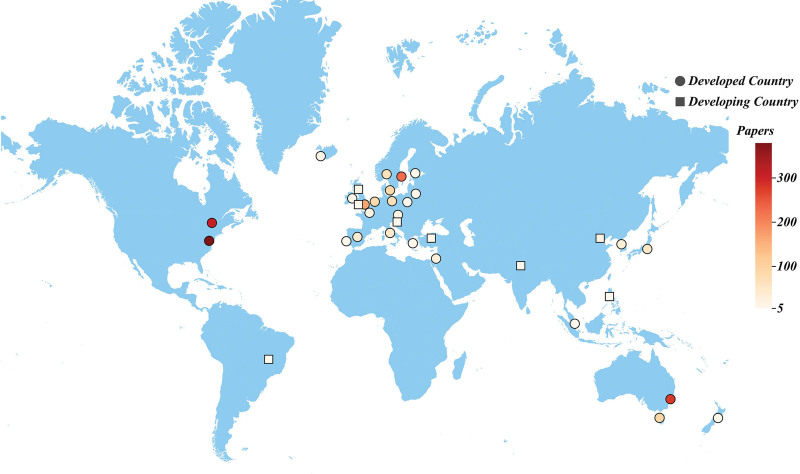
Distribution of countries with a publication volume >5.

**Figure 4.**
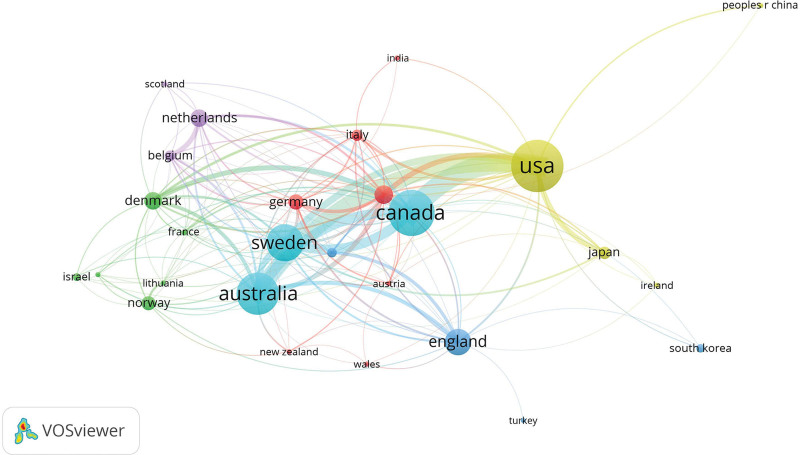
Visualization map of national research cooperation.

To evaluate the significant contributions of countries in the field of whiplash injury, this study analyzed the publication output of 57 countries. Table 1 showcases the top 10 countries ranked by the number of articles published. Remarkably, the United States emerged as the foremost contributor to whiplash injury research, with 379 articles, constituting 21.64% of all publications, and an average of 43.22 citations per article. Following closely, Canadian scholars contributed 309 articles (17.65% of all publications), with the highest average number of citations at 44.63.A visualization analysis was conducted using COOC for 23 countries that had published 10 articles or more, as depicted in Figures 3 and 4. Developed countries such as the United States, Canada, and Australia demonstrated not only high productivity but also close collaborative relationships. This underscores the potential for collaborative research to facilitate the advancement of other nations/regions. Additionally, the analysis revealed the formation of regional clusters based on geographic proximity.^[19]^

**Table 1:** Top 10 countries in the field of whiplash injuries research.

Serial number	Nationality	N	Serial number	Nationality	N
1	USA	379	6	Switzerland	92
2	Canada	309	7	Denmark	87
3	Australian	280	8	Netherlands	86
4	Sweden	229	9	Norway	65
5	England	166	10	Japan	57

N = number of publications.

**Figure 5.**
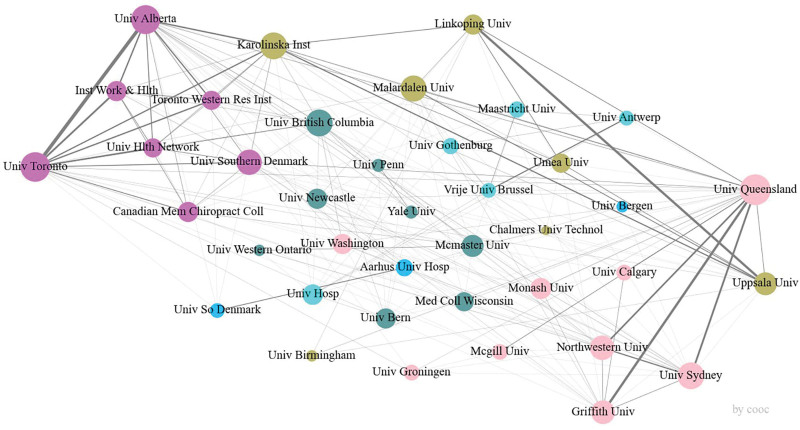
Visualization graph of organizational research cooperation.

The University of Queensland stands out as the most significant contributor to research on whiplash injury, accounting for 169 articles or 9.65% of the total. The top 10 institutions (refer to Table 2) are all based in the United States, Canada, Australia, and Sweden. The collaboration network among these institutions is shown in Figure 5.

**Table 2:** Top 10 research institutions in the field of whiplash injuries research.

Serial number	Institution	N	Serial number	Institution	N
1	Univ Queensland	169	6	Uppsala Univ	51
2	Univ Alberta	106	7	Karolinska Inst	50
3	Univ Toronto	75	8	Univ British Columbia	43
4	Univ Sydney	62	9	Yale Univ	39
5	Umea Univ	53	10	Linkoping Univ	38

N = number of publications.

Leading institutions in terms of publication output exhibit extensive and comprehensive coverage, with notable contributions from the University of Queensland and the University of Alberta. However, certain organizations demonstrate significant expertise in specific areas of research compared to others. For instance, the University of Alberta has emerged as a leader in studying whiplash injury resulting from accidents and in electromyography after WAD.^[20,21]^ On the other hand, the University of Queensland has pioneered research on dizziness symptoms and impairment in proprioception related to whiplash injury.^[22–24]^

**Figure 6.**
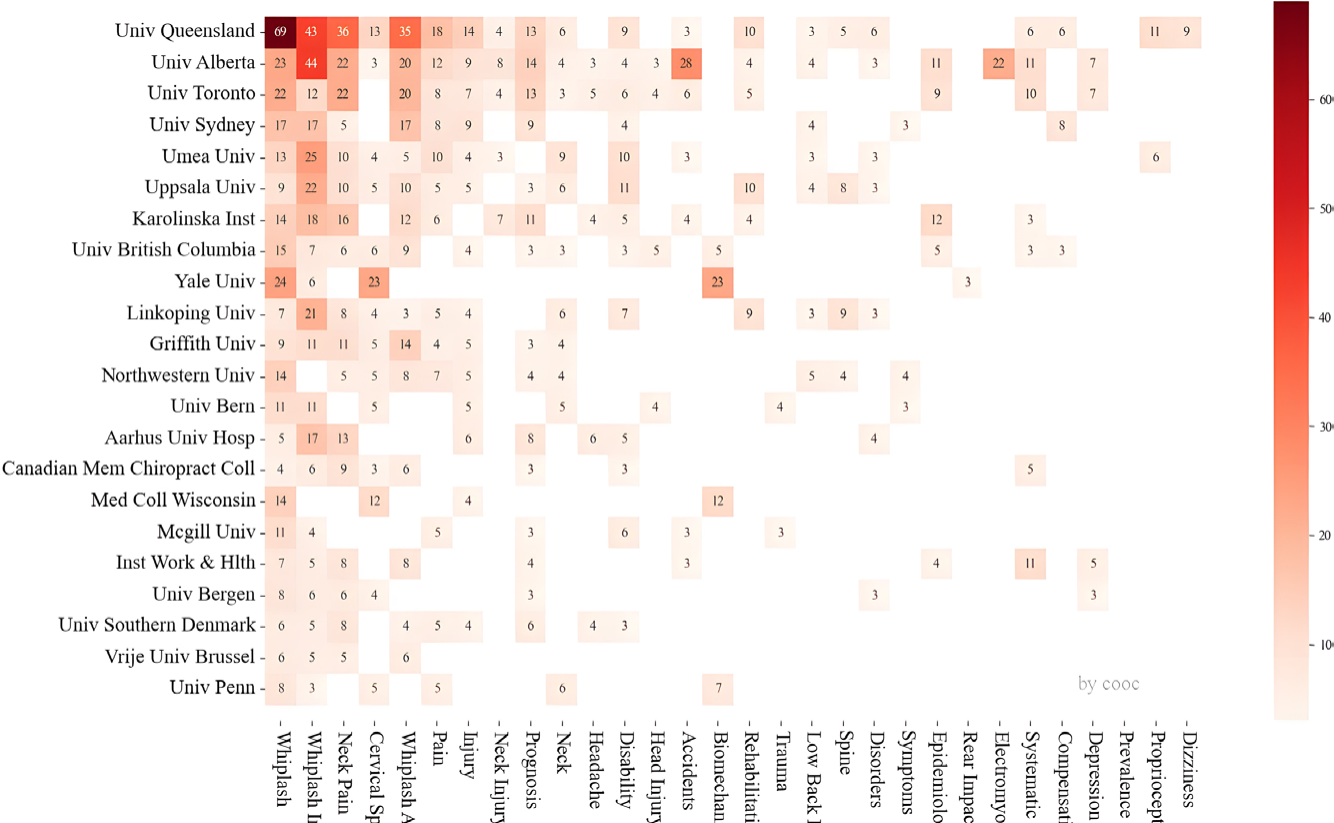
The second mock examination matrix of mechanism and keywords.

Yale University holds an absolute leading position in the field of spinal biomechanics research.^[25]^ Additionally, institutions such as the University of Linkoping and Uppsala University, along with the University of Queensland, are at the forefront of whiplash injury rehabilitation,^[26,27]^ and these 2 have conducted more in-depth research on exercise after whiplash injury (Fig. 6).^[28–32]^

### 3.4. Author situation analysis

**Figure 7.**
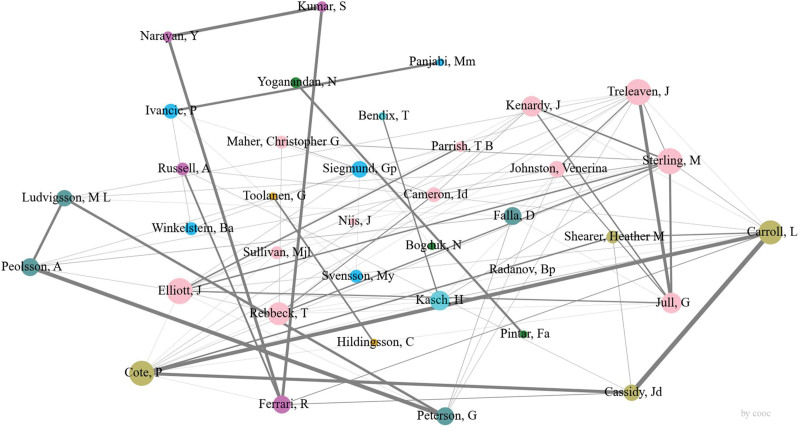
Visualization graph of researcher collaboration.

Between 1953 and 2022, a total of 4269 authors have contributed to studies on whiplash injury. Table 3 presents the top 10 most productive authors. Sterling, M from the University of Queensland was ranked first, with 94 articles that received 1074 citations and a mean number of citations of 11.43. Radanov, Bp from the University of Bern published 26 articles, receiving 729 citations, with an average citation count of 28.04, making him the scholar with the highest average citations in this field. To further investigate collaboration among authors in this area, we built a co-authorship network graph consisting of 38 authors who have published at least 10 articles (Fig. 7). The close collaboration observed among these researchers underscores the depth of cooperation within the field of whiplash injury research.

**Table 3:** Top 15 authors in the field of whiplash injury research.

Author	Country	Institution	N
Sterling, M^[33]^	Australian	Univ Queensland	94
Ferrari, R	Canada	Univ Alberta	63
Carroll, L	Canada	Univ Alberta	52
Jull, G	Australian	Univ Queensland	51
Cassidy, JD^[34]^	Canada	Toronto Western Hosp	50
Elliott, J	USA/Australian	Northwestern Univ/Univ Queensland	40
Cote, P	Canada	Univ Toronto	40
Treleaven, J	Australian	Univ Queensland	35
Peolsson, A^[31]^	Sweden	Linkoping Univ	32
Peterson, G	Sweden	Uppsala Univ/ Linkoping Univ	30
Ivancic, P	Sweden	Linkoping Univ	30
Kasch, H	Denmark	Aarhus Univ	26
Radanov, BP	Switzerland	Univ Bern	26
Panjabi, MM	USA	Yale Univ	25
Kumar, S	Canada	Univ Alberta	25

N = number of publications.

**Figure 8.**
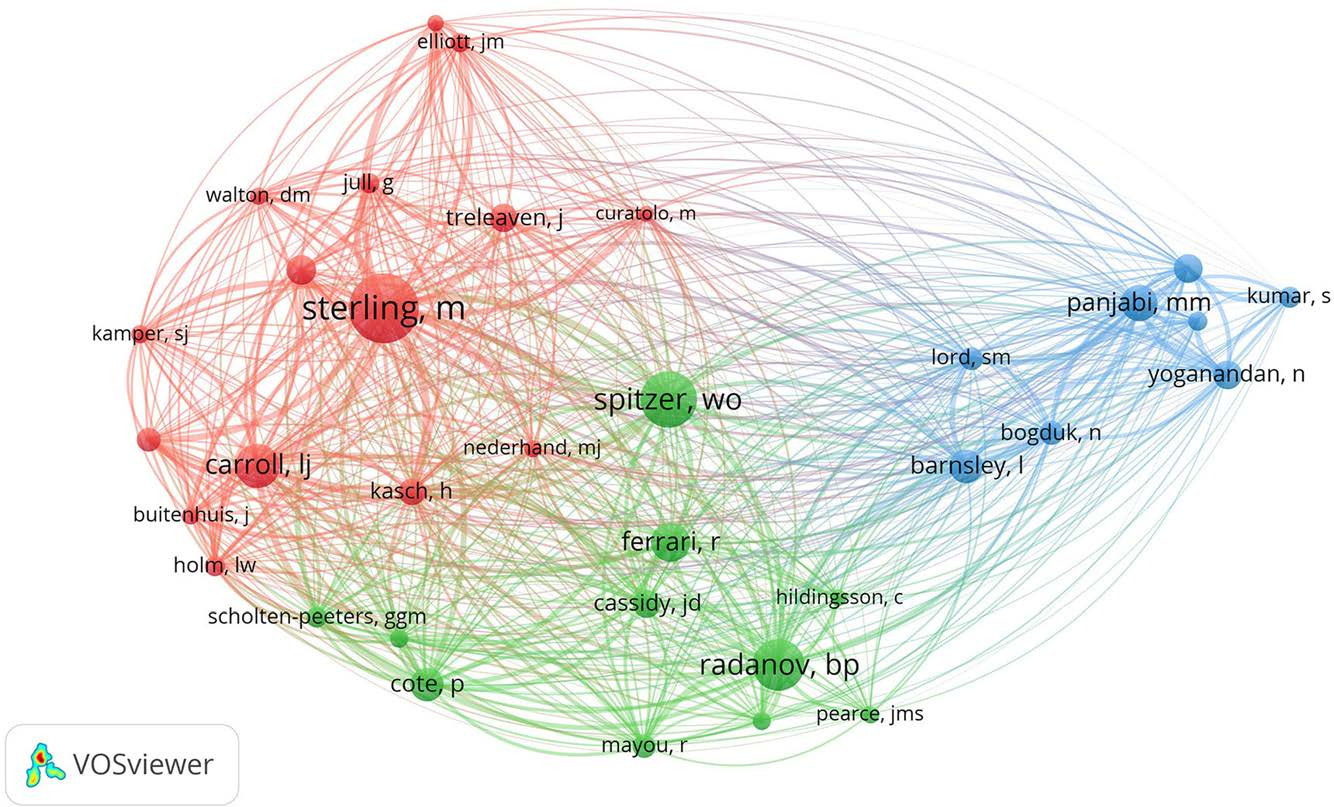
Author cited graph.

The citation count of authors serves as a vital metric of their impact within a particular field. Figure 8 depicts the co-citation network map of authors, encompassing 37 authors with at least 150 citations each. In this visualization, the size of the nodes reflects the total number of references, while the width of the edges indicates the connections between them. This co-citation network map offers valuable insights into the interconnections and collaborations among highly cited authors in the realm of whiplash injury research.

### 3.5. Analysis of journals and fields of study

From 1953 to 2022, a total of 450 journals have published at least one article on whiplash injury. Among these, the top 10 most productive journals collectively published 517 articles, representing approximately 29.53% of the total publications in this field. Table 4 outlines these top 10 journals, all of which boast JCR Q2 rankings and above. These journals cover a diverse range of research fields, including surgery, orthopedics, sports science, rehabilitation medicine, emergency medicine, clinical neurology, ergonomics, and transportation. The journal “Spine” leads with the highest number of publications, accounting for 8.11% of the total. Following closely, the “European Spine Journal” ranks second, with 58 articles published, equivalent to 3.33% of the total publications. The journal “Pain” holds the third position with 53 publications, amassing 4274 citations and the highest average citation count of 80.64 among the top 10 journals.

**Table 4:** Top 10 journals in the field of exercise therapy research for whiplash injury.

Journal name	N	%	NC	AC	JCR	IF
Spine	142	8.11%	6754	47.56	Q2	3.0
European Spine Journal	58	3.31%	1237	21.33	Q2	2.8
Pain	53	3.03%	4274	80.64	Q1	7.4
Clinical Journal of Pain	44	2.51%	939	21.34	Q2	2.9
Injury-International Journal of the Care of the Injured	41	2.34%	966	23.56	Q2	2.5
Accident Analysis and Prevention	41	2.34%	762	18.59	Q1	5.9
Manual Therapy	36	2.06%	770	21.39	Q2	2.3
Journal of Orthopedic & Sports Physical Therapy	36	2.06%	611	16.97	Q1	6.1
Bmc Musculoskeletal Disorders	33	1.88%	289	8.76	Q2	2.3
Journal of Manipulative and Physiological Therapeutics	33	1.88%	789	23.91	Q4	1.3

Manual therapy has been renamed musculoskeletal science and practice.

AC = average citation per article, IF = impact factor (2022), N = number of publications, NC = number of citations.

### 3.6. Analyzing cited references

Generally, the most frequently cited literature has the most influence on the scientific communities.^[35]^ Table 5 lists the top 10 most-cited articles in the field of whiplash injury. The research papers “Scientific monograph of the quebec task force on whiplash-associated disorders: Redefining ‘Whiplash’ and its management” and “The Neck Disability Index—A study of reliability and validity” are ranked first and second, respectively, with citations of 235 and 233.^[3,36]^ Notably, all of the top 3 articles have received over 200 citations. “Course and Prognostic Factors for Neck Pain in Whiplash-Associated Disorders (WAD)” holds the highest average annual citation count among the ten articles, further highlighting its significant influence within this field.^[37]^

**Table 5:** Top 10 cited literature in the field of whiplash injuries.

Article title	First author	PY	NC	AC
Scientific Monograph of the Quebec Task Force on Whiplash-Associated Disorders: Redefining “Whiplash’’ and Its Management	Spitzer, WO^[3]^	1995	235	8.39
The Neck Disability Index—A Study of Reliability And Validity	Vernon, H^[36]^	1991	233	7.28
Effect of Eliminating Compensation for Pain and Suffering on the Outcome of Insurance Claims for Whiplash Injuries	Cassidy, JD^[34]^	2000	224	9.74
Whiplash injuries	Barnsley, L^[1]^	1994	198	6.83
Long-Term Outcome after Whiplash Injuries: A 2-Year Follow-Up Considering Features of Injury Mechanism and Somatic, Radiologic, and Psychosocial Findings.	Radanov, BP	1995	175	6.25
Course and Prognostic Factors for Neck Pain in Whiplash-Associated Disorders (WAD)	Carroll, LJ	2008	167	11.13
Prognostic factors of whiplash-associated disorders: a systematic review of prospective cohort studies.	Scholtenpeeters, GGM	2003	150	7.50
Course And Prognostic Factors of Whiplash: A Systematic Review and Meta-Analysis	Kamper, SJ^[37]^	2008	137	9.13
The prognosis of neck injuries resulting from rear-end vehicle collisions.	Norris, SH	1983	135	3.38
The development of psychological changes following whiplash injuries	Sterling, M	2003	130	6.50

AC = average number of citations per year, NC = number of citations, PY = publication year.

Table 5 highlights the prevalence of articles focusing on the risk factors associated with WAD. Interestingly, the majority of these articles were published approximately 20 years ago, indicating the pivotal role of classic literature in this research field. Furthermore, half of these articles are review articles, providing a comprehensive overview of the current state of research on WAD. This suggests that WAD was extensively explored 2 decades ago. From these articles, several key points emerge: Firstly, the Quebec Task Force represents the first comprehensive guide on the management of WAD.^[3]^ Secondly, the Neck Disability Index not only offers an effective tool for assessing neck pain but also has been validated for whiplash injuries.^[36]^ Additionally, insurance claims programs are crucial aspects of WAD management, as they are closely linked to accidents. Numerous policies have been implemented to mitigate the costs associated with compensation.^[34]^

### 3.7. Analysis of keyword co-occurrence

**Figure 9.**
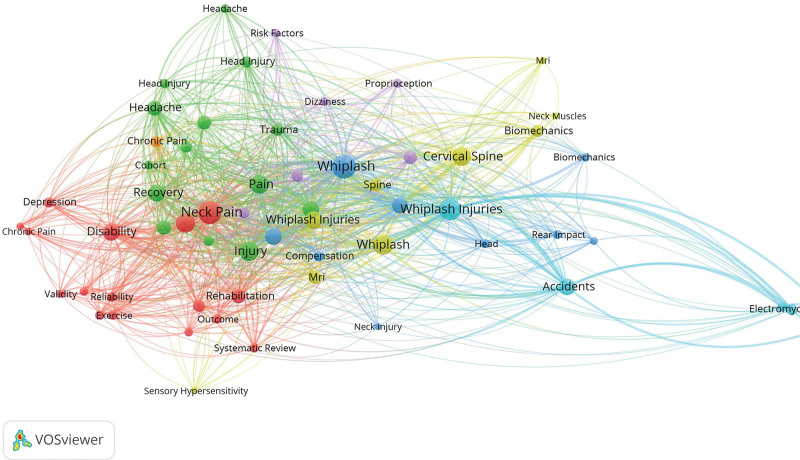
Keyword clustering diagram.

Keywords serve as succinct summaries of the core concepts within a paper, and keyword co-occurrence analysis can unveil the hotspots of a research field. By employing COOC and VOSviewer to visualize keyword co-occurrence networks for 1685 documents, 60 keywords with a maximum or equal frequency of 13 were selected for visualization (Fig. 9). For a more detailed understanding of specific keywords, high-frequency terms with a frequency exceeding 40 were highlighted in Table 6. From the table, it is evident that “whiplash” is the most frequently occurring term, appearing 538 times. “Whiplash,” “whiplash injury,” “neck pain,” “cervical spine disease,” and “whiplash-associated disorders” are high-frequency keywords that represent the core concepts in this field.

**Table 6:** High-frequency keywords.

Serial number	Keywords	N	Serial number	Keywords	N
1	Whiplash	538	11	Disability	91
2	Whiplash Injuries	437	12	Head Injury	89
3	Neck Pain	238	13	Accidents	86
4	Cervical Spine	185	14	Headache	84
5	Whiplash Associated Disorders	184	15	Biomechanics	80
6	Pain	135	16	Mri	74
7	Injury	131	17	Recovery	73
8	Neck Injury	112	18	Chronic Pain	61
9	Prognosis	107	19	Rehabilitation	56
10	Neck	96	20	Trauma	47

N = number of publications.

Indeed, words like “disability” and “rehabilitation” have been central to research in the field over the past seventy years. Many patients experience short-term symptoms, such as pain, and long-term dysfunction, like headaches and dizziness, which necessitate active rehabilitation interventions. Early physical therapy interventions have been shown to significantly improve dysfunction and prognosis.^[38,39]^ Concurrently, there is a growing body of research investigating the mechanisms underlying WAD. Scholars are delving into the biomechanics of the cervical spine during car accidents, exploring the impact of various collision angles and directions on whiplash injuries. Additionally, studies are examining how different car seat designs and headrest configurations influence the occurrence and severity of whiplash injuries. Various collision factors, such as speed and direction, have been shown to influence the severity and prognosis of these injuries, a fact effectively demonstrated through electromyography.^[40]^

## 4. Discussion

### 4.1. An overview of whiplash injury research

This article is the first bibliometric study to analyze global trends in whiplash injury research. The number of publications and citations in this field indicates an increasing interest in whiplash injury since the 20th century. Notably, over the past 2 decades, there has been a consistent annual publication rate exceeding 35 articles. This trend is intricately linked to the widespread utilization of vehicles, reflecting the increasing societal awareness and concern regarding whiplash injuries resulting from motor vehicle accidents.

Collaboration is also a main feature in the field of whiplash injury research, and cross-national and cross-institutional collaborations are common in this research domain. which contribute to advancing the research field, providing valuable collaboration and consultation information. The author’s collaborative research is related to the high citation rate.

Research on whiplash injury has gained recognition and dissemination in top journals across various disciplines, spanning rehabilitation science, sports science, and ergonomics.^[33,41–43]^ The characteristic feature of cross-disciplinary integration is evident in this field, as whiplash injury may involve multiple disciplines, including emergency medicine, spinal science, rehabilitation medicine, neurology, biomechanics, and engineering.^[44,45]^ Multidisciplinary collaboration is therefore emphasized as a key aspect of WAD management, highlighting the necessity for a comprehensive approach to address the multifaceted nature of whiplash injury and its associated challenges.

A comprehensive medical history inquiry and physical examination are imperative during the initial visit to the emergency department, particularly to evaluate for red-flag symptoms and severe conditions. It is recommended that emergency department physicians utilize clinically validated rules to manage patients’ relevant conditions, including the practice guidelines established by the Quebec Task Force in 2010 and the clinical guidelines introduced by the Australasian College for Emergency Medicine and the Australian Society for Emergency Medicine in 2018.^[46]^ This approach not only ensures patients receive the most scientifically appropriate treatment but also maximizes cost savings on examinations.^[47]^ In cases of severe injuries such as fractures, dislocations, or neurological impairments of the cervical spine, immediate referral for surgical intervention should be pursued.^[48]^ Once serious pathology has been ruled out, active interventions should be taken to alleviate symptoms. These measures may include medication, exercise prescription, health promotion, physiotherapy, among others.^[3]^ Furthermore, the severity of WAD may be influenced by engineering factors such as car seat height, headrest design, and crash speed, in addition to individual factors such as age, gender, and the degree of previous neck pain.^[49]^ Therefore, a comprehensive approach that considers both individual and environmental factors is essential in managing WAD effectively.

### 4.2. Research hotspots and frontiers of whiplash injury

Analysis of the cited literature reveals the most influential articles that have laid the foundation of knowledge for future research.^[50]^ For instance, the standardization of whiplash injury treatment began with the formation of the Quebec Task Force in 1995 and the publication of the first clinical guideline related to whiplash injury.^[3]^ The emergence of the Neck Disability Index provides a quantitative opportunity for the assessment of whiplash injury.^[36]^ Through analysis of the cited literature, several clusters have been identified in this research field: (1) insurance claims related to whiplash^[51]^; (2) prognosis of whiplash and its influencing factors; and (3) management of whiplash.^[11,52]^ Among these, the identification of risk factors influencing the prognosis of whiplash has always been a top priority in this research field. This highlights the ongoing efforts to enhance understanding and management strategies for whiplash injury.

Research in the insurance field has revealed a correlation between the types of insurance claim policies and the occurrence rate as well as the recovery time of WAD. It has been observed that more complex insurance claim procedures or stricter claim conditions are associated with a lower incidence rate of WAD. Furthermore, claimants tend to experience faster recovery if compensation is not obtained.^[53]^ This underscores the influence of insurance policies and claim procedures on the occurrence and management of WAD, highlighting the importance of considering insurance-related factors in research and clinical practice within this field.

Understanding the prognostic factors of WAD is crucial for developing effective management and intervention strategies, as well as designing reimbursement policies to alleviate the burden of WAD.^[37]^ Prognostic factors can directly influence the resolution of neck pain and associated symptoms, as well as the timeframe for their recovery. Factors of WAD include demographic and socioeconomic factors, prior health conditions or pain or comorbidities, collision factors, initial symptoms, psychosocial factors, genetic factors, and early interventions adopted. Linda. J et al found that precrash self-factors rarely affect prognosis and that posttraumatic factors affect prognosis more. For example, factors related to the collision itself (e.g., collision orientation, headrest type) were largely unpredictable; however, posttraumatic psychological factors and early and frequent health-care use were associated with poorer recovery.^[37]^ David M et al found that preinjury neck pain and collision factors can affect the prognosis of WAD. In addition, a history of headaches before the accident, rear-end collision history, and older age were also identified as factors associated with poorer prognosis.^[54]^ Therefore, collecting more data to analyze potential factors is essential for building a more comprehensive clinical prediction model.^[55,56]^

During the rehabilitation of WAD, the Quebec Task Force and issues related to neck pain and disability are frequently discussed.^[2,38,57]^ Pain emerges as the most prevalent symptom post-whiplash injury, and alleviating pain stands as the primary treatment objective.^[41]^ Joint stiffness and limited mobility are common functional impairments in patients with whiplash injury,^[58]^ which resolves in most patients with conservative treatment.^[59]^ Recent research suggests that patients classified as Grade II may exhibit elevated sensory thresholds and other signs, potentially challenging the Quebec Classification System.^[60]^ Nevertheless, the significance of clinical decision-making remains undeniable in managing WAD effectively.

**Figure 10.**
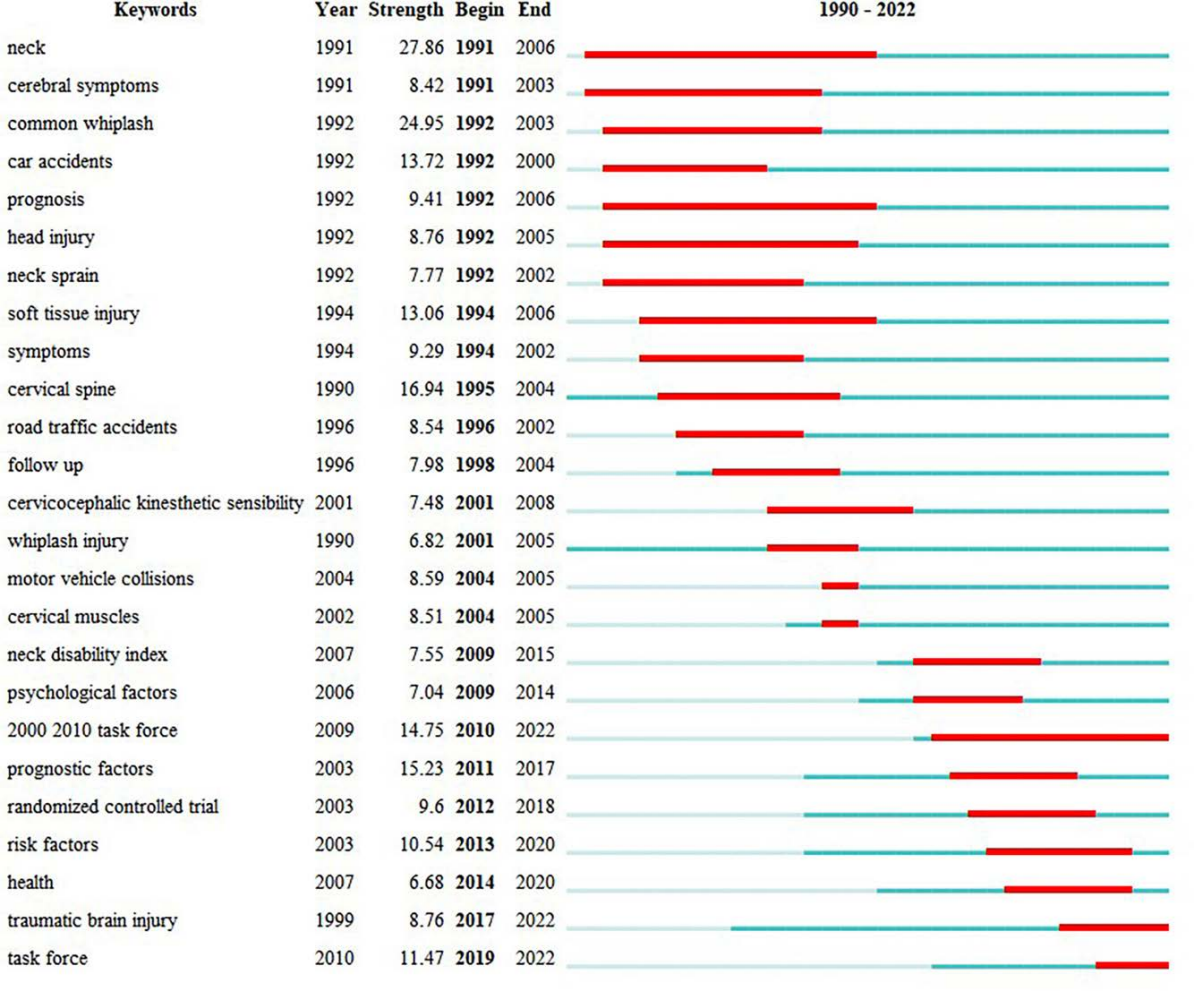
Graph of burst words.

To further clarify the sudden emergence of research hotspots in the field of whiplash injury, the Burstiness analysis function of CiteSpace was further used, and the results are shown in Figure 10. The Burstiness words were observed to be divided by 2009, indicating a shift in research focus. Prior to 2009, research predominantly centered on whiplash injury and its associated symptoms.^[61–63]^ Subsequently, after 2009, there was a notable surge in research interest surrounding the standardized management of whiplash injury, particularly with the advent of the Quebec Task Force between 2000 to 2010, which significantly contributed to the advancement of whiplash injury management once again.^[64]^ Notably, traumatic brain injury and mental health issues such as depression and anxiety were identified as potential consequences of whiplash injury.^[65,66]^ Grimmelt et al demonstrated in animal experiments that whiplash injury sustained from car accidents lead to an increase in extracellular concentrations of glutamate, aspartate, and γ-aminobutyric acid. Neuroimaging reveals characteristic changes indicative of traumatic brain injury. This underscores the evolving landscape of whiplash injury research and the growing recognition of its multifaceted impacts on individuals’ health and well-being.^[67,68]^

Overall, while certain research directions have garnered significant attention in the past, some areas have experienced a decline in recent years. For example, there has been a decrease in studies focusing on insurance claims related to whiplash injuries.^[69]^ Conversely, research on risk factors has remained a persistent focus, as few studies have comprehensively summarized all relevant risk factors.^[70]^ Thus, there is ample opportunity for more concise investigations in this area. The treatment of WAD has consistently been a primary research focus due to its significant impact on patient prognosis. With advancements in technology, new techniques are continuously being applied to enhance the management of WAD.^[52]^

One limitation of this study is its exclusive reliance on the WoSCC database, which may potentially overlook relevant research published in other databases or sources. Additionally, certain features of the software tools utilized, such as VOSviewer and Citespace, are only applicable for analyzing data from 1990 onwards, thus limiting the scope of historical analysis. However, despite these limitations, the strength of this study lies in its comprehensive bibliometric analysis of the field of whiplash injury. By employing a combination of 3 software tools, the study offers a robust and unparalleled examination of research trends, collaborations, and hotspots within the field. This comprehensive approach enhances the reliability and validity of the findings, providing valuable insights for researchers, practitioners, and policymakers alike.

## 5. Conclusion

This study systematically summarized the articles on whiplash injury research from 1953 to 2022 through bibliometric analysis. The findings highlight the concern and interest in a multidisciplinary approach to the study of whiplash injury. The United States, Australia, and Canada are leaders in the field of whiplash injury research, with the University of Queensland playing a central role in collaborative research. The cited literature primarily focuses on insurance claims, prognosis, and influencing factors and the management of WAD. “Validated clinical decision rules” and “neurological disorders” have received particular attention in recent years. The analysis also identifies emerging research frontiers, including “psychological functional impairment,” “prognostic factors of whiplash injury,” “clinical prediction rule models,” “mild traumatic brain injury,” “physiotherapy” and “central sensitization.” Examining the patterns and trends of whiplash injury research, valuable insights are provided to better understand the current research status and may inform future research directions.

## Author contributions

**Conceptualization:** Yaqi He, Yang Liu.

**Data curation:** Yaqi He, Jinpeng Gao.

**Formal analysis:** Yaqi He.

**Funding acquisition:** Jinghua Qian.

**Investigation:** Yaqi He.

**Methodology:** Yaqi He, Jinpeng Gao, Yang Liu, Jinghua Qian.

**Project administration:** Yaqi He.

**Resources:** Jinghua Qian.

**Software:** Yaqi He, Jinpeng Gao.

**Supervision:** Yang Liu, Jinghua Qian.

**Visualization:** Jinpeng Gao, Yang Liu.

**Writing – original draft:** Yaqi He.

**Writing – review & editing:** Yaqi He, Yang Liu, Jinghua Qian.
